# Selenium and Type 2 Diabetes: Systematic Review

**DOI:** 10.3390/nu10121924

**Published:** 2018-12-05

**Authors:** Lindsay N. Kohler, Janet Foote, Connor P. Kelley, Ana Florea, Colleen Shelly, H-H Sherry Chow, Paul Hsu, Ken Batai, Nathan Ellis, Kathylynn Saboda, Peter Lance, Elizabeth T. Jacobs

**Affiliations:** 1 Department of Health Promotion Sciences, Mel and Enid College of Public Health, University of Arizona, Tucson, AZ 85724, USA; 2 Department of Epidemiology, Mel and Enid College of Public Health, University of Arizona, Tucson, AZ 85724, USA; jfoote@email.arizona.edu (J.F.); cpkelley@email.arizona.edu (C.P.K.); aflorea@email.arizona.edu (A.F.); pchhsu@email.arizona.edu (P.H.); jacobse@email.arizona.edu (E.T.J.); 3 Department of Medicine, University of Arizona, Tucson, AZ 85724, USA; plance@uacc.arizona.edu; 4 Department of Epidemiology, Harvard T.H. Chan School of Public Health, Boston, MA 02115, USA; colleenshelly@gmail.com; 5 Department of Medicine, University of Arizona Cancer Center, Tucson, AZ 85724, USA; schow@email.arizona.edu (H.-H.S.C.); naellis@email.arizona.edu (N.E.), ksaboda@email.arizona.edu (K.S.); 6 Department of Surgery, University of Arizona, Tucson, AZ 85724, USA; kbatai@email.arizona.edu

**Keywords:** type 2 diabetes, selenium, selenium supplementation, glucose, insulin resistance, review

## Abstract

Several studies have investigated the potential role of selenium (Se) in the development of type 2 diabetes (T2D) with disparate findings. We conducted a systematic review and meta-analysis to synthesize the evidence of any association between Se and T2D. PubMed, Embase, and Scopus were searched following the Preferred Reporting Items for Systematic Reviews and Meta-analysis Approach (PRISMA). Sixteen studies from 15 papers met inclusion criteria defined for this review. Of the 13 observational studies included, 8 demonstrated a statistically significant positive association between concentrations of Se and odds for T2D, with odds ratios (95% confidence intervals) ranging from 1.52 (1.01–2.28) to 7.64 (3.34–17.46), and a summary odds ratio (OR) (95% confidence interval (CI)) of 2.03 (1.51–2.72). In contrast, among randomized clinical trials (RCTs) of Se, a higher risk of T2D was not observed for those who received Se compared to a placebo (OR = 1.18, 95% CI 0.95–1.47). Taken together, the results for the relationship between Se and T2D differ between observational studies and randomized clinical trials (RCTs). It remains unclear whether these differences are the result of uncontrolled confounding in the observational studies, or whether there is a modest effect of Se on the risk for T2D that may vary by duration of exposure. Further investigations on the effects of Se on glucose metabolism are needed.

## 1. Introduction

The trace element selenium (Se) gained momentum as a potential chemopreventive agent with the publication of the results of the Nutritional Prevention of Cancer (NPC) trial in 1996 [1]. In this randomized, double-blind chemoprevention trial, participants were supplemented with either 200 µg Se per day as brewer’s yeast or a matched placebo [1]. No benefit of Se supplementation was observed for the primary endpoint of the trial, non-melanoma skin cancer. However, secondary analyses revealed a statistically significant 58% reduction in colorectal cancer incidence and a 63% reduction in prostate cancer for those in the Se supplementation arm compared to those who received the placebo [1] and a longer-term follow-up demonstrated that this effect was attenuated [2]. 

The primary NPC results were followed by the work of Stranges et al., which showed that participants in the trial who were supplemented with Se had a statistically significantly higher rate of type 2 diabetes (T2D) than those in the placebo group (HR (95% CI) of 1.55 (1.03–2.33)) [3]. Furthermore, those in the highest tertile for blood Se levels at baseline before administration of Se supplementation had a significantly higher risk of T2D than those with lower baseline Se levels (HR (95% CI) of 2.70 (1.30–5.61)) [3]. These results were unexpected given that Se was first hypothesized to potentially reduce the risk for insulin resistance and T2D given that the treatment of mice with selenate, an inorganic form of Se, resulted in insulin-like effects and mitigated insulin resistance [4,5]. In contrast to studies with rodent models, human studies have tended to focus on proteins that incorporate the amino acids selenomethionine and selenocysteine [6]. Glutathione peroxidase-1 (GPx-1) is a selenoprotein that has garnered a great deal of attention due to its beneficial ability to scavenge reactive oxygen species and prevent oxidative damage [6,7,8]. However, studies in rodent models have indicated that there is a potentially harmful effect of GPx-1. When it is overexpressed, it causes hyperglycemia; GPx-1 knockout mice have better glucose homeostasis compared to wild-type animals [9,10]. These findings may also suggest that a key feature of Se in glucose homeostasis depends on a balance of meeting Se nutritional requirements and avoiding excess exposure. Thus, since the publication of the results of the NPC trial related to Se and T2D, several later studies also assessed this association, with equivocal results.

The aim of the present study was, therefore, to conduct a review of the literature to evaluate and synthesize the evidence from studies on the association between selenium and T2D. 

## 2. Materials and Methods 

### 2.1. Methods

#### Search Strategy and Identification of Studies

The protocol was registered with PROSPERO, an international prospective register of systematic reviews (Ref: CRD42018081073). We employed the preferred reporting items for systematic reviews and meta-analysis approach (PRISMA) guidelines to conduct the systemic review [11]. We used the search terms “Selenium and type 2 diabetes”, “selenium”, “diabetes”, “glucose”, and “selenium supplementation” in the PubMed, Embase, and Scopus databases in November 2017. We restricted the search to include only human studies, English-language publications, and full-text articles from the past 15 years. We saved the PubMed search parameters and re-ran the query each month to identify and include new publications, and further checked each eligible article for references that may have been missed through the search.

Two reviewers (L.N.K. and A.F.) reviewed all identified titles and abstracts from the studies identified initially and a data extraction form was developed that included the first author’s name, title, year of publication, study population, study design, length of follow-up period, study endpoints (development of T2D; glucose levels), and the point estimates of the original publication. Bias was assessed in all included studies using The Critical Appraisal Skills Programme’s: Making Sense of Evidence [12]. Parameters included in the evaluation of bias were recruitment procedures, assessment technique for the exposure, outcome and any potential confounders, and generalizability of the work. Any discordant selections of studies for inclusion were resolved by a third reviewer (E.T.J.).

### 2.2. Inclusion and Exclusion Criteria

Any observational study published within the past 15 years with at least 100 participants that evaluated Se supplementation or measured Se status with T2D as an outcome was included; while all clinical trials with data available for both the numerator and denominator were included. The minimum criteria for inclusion were (1) Se supplementation or measured levels in whole blood, serum or plasma, or estimates of dietary intake of Se obtained from food frequency questionnaires; and (2) a reported outcome of T2D. Type 2 diabetes was defined at follow-up by self-report or measured by fasting blood sugar >126 mg/dL on two separate tests or a glucose level of >200 mg/dL after two hours during an oral glucose tolerance test (OGTT). Articles written in languages other than English were excluded as they could not be adequately reviewed by the research team. In addition, commentaries that did not present original data were excluded from the present analysis. Authors of potentially eligible published reports that lacked key information, such as the number of cases and controls, were contacted for the missing information. If this information was not forthcoming, various ranges of the T2D incidence rate for the lowest Se quantile, including the average of the T2D incidence rate among the studies with information available, and the reported odds ratio were used to derive the number of cases and controls for both the lowest and highest Se quantiles. This sensitivity analysis strategy indicated how sensitive the summary statistic was to those studies.

### 2.3. Statistical Analysis

Weighted summary estimates and 95% confidence intervals (CIs) were obtained by random-effects meta-analysis by study design, which accounts for the between-study variation. For studies that did not report the number of type 2 diabetes cases in both the lowest and highest quantiles, an average prevalence of type 2 diabetes for the lowest quantile group from other studies was calculated. A sensitivity analysis was conducted with varying T2D prevalence rates (5–10%), based on other studies in this review that provided prevalence information. Cochran’s Q was used to examine the presence of heterogeneity of treatment effects across included studies (*p* < 0.1). Further, the *I^2^* statistic was examined to quantify the presence of heterogeneity, with a value >50% indicating significant heterogeneity. Publication bias was assessed via funnel plot visual inspection, while the meta-analysis was performed using the R package “metafor” (R version 3.5.0, Vienna, Austria). 

## 3. Results

**Figure 1:**
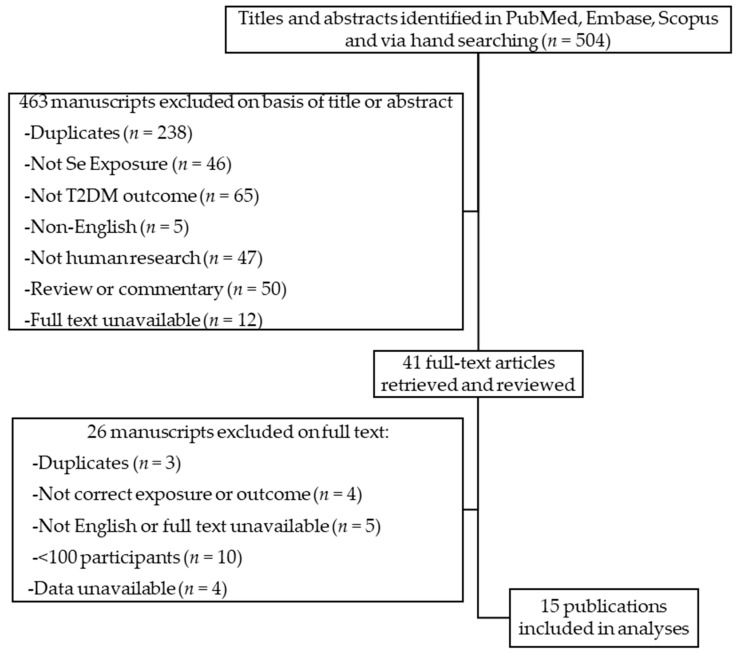
Manuscript selection flowchart.

As shown in Figure 1, a total of 504 potentially relevant studies were reviewed; after the removal of duplicates and exclusion on the basis of title or abstract, 41 full papers evaluating Se and T2D were retained for in-depth consideration.

We identified 15 manuscripts that met the a priori criteria for inclusion. These are described in more detail in Table 1. These studies represent analyses of data from observational studies, as well as supplementation trials. All trials and observational studies mentioned fulfillment of ethics board approval criteria, except for Laclaustra et al. [13] and Bleys et al. [14], both of which utilized data from the National Health and Nutrition Examination Survey (NHANES), a publicly available dataset. 

### 3.1. Observational Studies

The three longitudinal studies that were included followed a total of 9352 participants. The Uppsala Longitudinal Study of Adult Men (ULSAM, *n* = 936 men) [15] included 20 years of follow-up, while the HORmones and Diet in the ETiology of Breast Cancer Study (ORDET, *n* = 7182 women) [16] followed participants for 16 years. The third cohort was from the Hortega Study and included both men and women (*n* = 1234) and followed for an average of 13.2 years [17]. The ORDET study investigated dietary and/or supplemental selenium intake via baseline food frequency questionnaires, while the ULSAM study utilized baseline serum and the Hortega study, plasma [15,16,17]. Five case–control studies met inclusion and exclusion criteria, altogether comprising 1330 cases and 3664 controls [18,19,20,21,22]. All five studies evaluated biochemical selenium status via plasma (*n* = 1) [18], serum (*n* = 1) [21], or whole blood (*n* = 3) [19,20,22]. There were also five cross-sectional studies included, with a total of 18,382 participants, published between 2007 and 2018 [13,14,17,23,24]. Wei et al. investigated dietary and/or supplemental selenium intake via food frequency questionnaires [24]. The remaining four cross-sectional studies evaluated biochemical selenium status via plasma (*n* = 2) [17,23] or serum (*n* = 2) [13,14].

### 3.2. Randomized Controlled Trials

We included three randomized clinical trials (RCTs), with a combined total of 20,290 participants, in this review [3,25,26]. All three trials assessed cancer or precancerous lesions as a primary outcome; however, the Selenium and Vitamin E Cancer Prevention Trial (SELECT) [25] (*n* = 17,448) enrolled only men, while the NPC trial (*n* = 1202) and the Selenium Trial (*n* = 1640) included men and women [3,26]. For each trial, investigators randomized participants to receive a 200 µg/day Se supplement as either selenized yeast [3,26] or *L*-selenomethionine [4] or a matched placebo for an average of 5.4 years (range 3.0 to 7.7 years).

### 3.3. Risk Assessment and Heterogeneity

**Figure 2:**
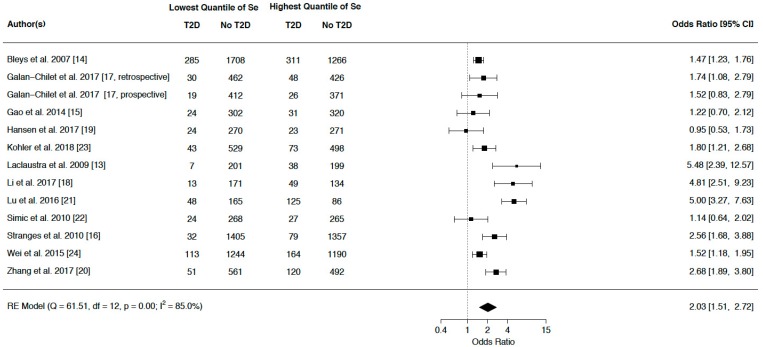
Odds ratios (95% CIs) and summary statistics for the observational studies.

We evaluated ORs of highest versus lowest quantile of Se level for observational studies and Se level in Se intervention versus placebo groups for RCTs to assess the association between Se exposure and risk of T2D. As shown in Figure 2**,** observational study participants in the highest Se quantiles had a statistically significant increased odds of T2D compared to those in the lowest quantiles (pooled OR = 2.03, 95% CI 1.51–2.72). 

**Figure 3:**
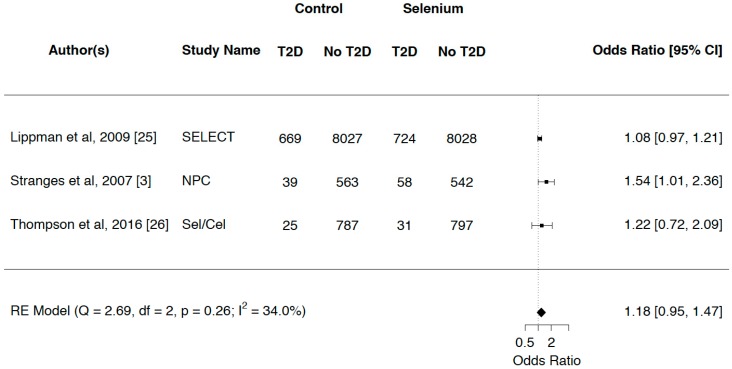
Odds ratios (95% CIs) and summary statistics for randomized clinical trials (RCTs).

Considerable heterogeneity was observed in the observational studies (*p* = 0.00, *I*^2^ = 85.0%). Figure 3 demonstrates that for RCTs, there was a nonsignificant increased risk of T2D between those who received a Se supplement and those who were assigned placebo, with a pooled OR (95% CI) of 1.18 (0.95–1.47). Moderate, nonsignificant heterogeneity was seen among the RCTs (*p* = 0.26, *I*^2^ = 34.0%).

**Figure 4:**
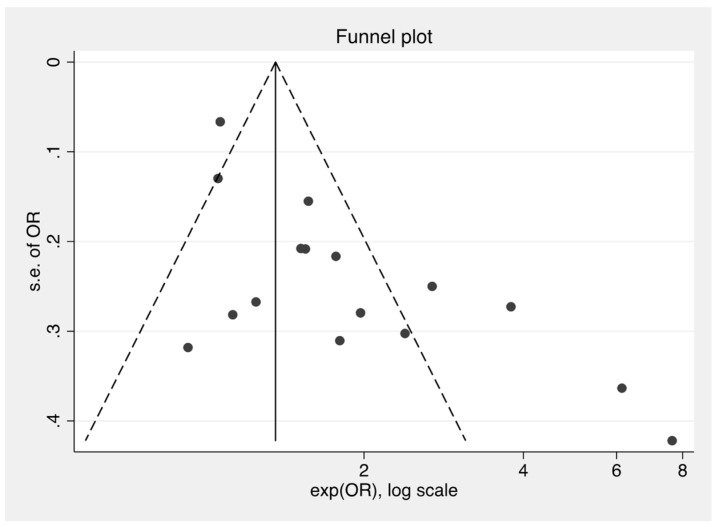
Funnel plot for publication bias.

Figure 4 demonstrates a funnel plot for the treatment effect (log scale) plotted against the study size, as measured by the standard error of the treatment effect. The tendency toward asymmetry indicates evidence for publication bias.

## 4. Discussion

The results of this meta-analysis of Se and T2D showed that in RCTs, there is no significant effect of Se on the incidence of T2D. However, observational studies demonstrate a positive association between blood levels of Se and odds for prevalent T2D. Reconciliation of these findings is challenging, but there are several potential explanations for the differences in the clinical trial results compared to those from the observational studies.

Results from preclinical studies led to the further study of Se and glucose metabolism in large epidemiological studies in human populations. The work of Stranges et al. showed that within the NPC trial in which participants were followed for more than seven years, there was a statistically significant increase in T2D incidence among those who received Se compared to a placebo, with a hazard ratio and 95% confidence interval of 1.55 (1.03–2.33) [3]. These results were published while two additional large trials of Se supplementation were underway in the United States: SELECT and the Selenium Trial [25,26], neither of which showed an overall statistically significant effect of Se supplementation on incident T2D. The Selenium Trial did report a significantly increased risk for T2D among the study participants who received Se and were older than 63 years of age [26]. Results of our previously published cross-sectional analyses of the Selenium Trial showed that baseline levels of Se were associated with a significantly higher odds of prevalent T2D, but that there was no age-related increase in risk of T2D [23]. These findings mirror those in the present meta-analysis of observational studies, as well as those of others [27], whereby a significantly increased risk for T2D was found among those with higher blood levels of Se [13,14,23,24].

The largest observational study to date employed NHANES data to examine whether Se was related to T2D risk in a US study population of more than 8000 individuals [13]; those in the highest quantile for Se concentrations had an OR (95% CI) of 1.57 (1.16–2.13) [13] for T2D compared to the lowest. In an Italian study population, Stranges et al. reported that the proportion of T2D cases in the highest tertile of baseline Se concentrations was significantly higher than those in the lowest tertile [28], and in a study conducted in China, individuals in the highest quartile of blood Se levels had a significantly higher odds of T2D compared to the lowest tertile, with an OR (95% CI) of 2.69 (1.31–3.49) [20]. In contrast, in two studies conducted within the Nord-Trøndelag Health Survey (HUNT-3) in Norway, no significant association was found [19,22]. Overall, we identified a statistically significant direct relationship between Se and T2D in observational studies, but not among RCTs. The summary odds ratio (95% CI) calculated in the present study of 1.18 (0.95–1.47) is similar to that of another group that included RCTs with small numbers of participants for their estimates, and which reported a statistically significant finding of 1.11 (1.01–1.22) [27]. Hence, the effect of selenium on T2D in RCTs remains equivocal, while those of observational studies appear to be more consistent.

The differences in the results from the observational studies and RCTs could arise from several different factors. The first is related to causality, which cannot be ascertained from observational studies. As shown in the current work, results from clinical trials provide scant evidence of a causal effect of Se on the development of T2D. It would be expected that if there were a notable impact of selenium on glucose dysregulation, it would likely be observed in the clinical trials that occurred after the NPC trial; notably, the SELECT and Selenium Trials [25,26]. These two trials used different intervention agents, with SELECT employing selenomethionine and the Selenium Trial, like the NPC trial, using selenized yeast. As reviewed by Rayman and Stranges, selenomethionine cannot be used directly for selenoprotein synthesis after consumption and must undergo catabolism first [7]. Experimental models with yeast also indicate that the effects of selenomethionine may vary depending on the presence of sulfur compounds that may compete with selenomethionine uptake [29]. In contrast, the Selenium Trial employed selenized yeast, which is the same agent that was shown in the work by Stranges et al. to be associated with a higher risk for T2D in the NPC trial [3]. Selenized yeast contains various amounts of selenomethionine in addition to numerous other selenium compounds of varying concentration and activity [30], highlighting the possibility that a selenium species other than selenomethionine might cause impaired insulin sensitivity or glucose metabolism. However, the Selenium Trial only demonstrated an increased risk for T2D for those at least 63 years of age at randomization, not overall (HR = 1.25, 95% CI 0.74–2.11), among those who received the supplement, indicating that selenized yeast does not appear to exert effects on T2D that are greater than selenomethionine [26]. 

Rayman and Stranges suggested another possibility, which was that participants in the NPC trial were recruited due to the presence of nonmelanoma skin cancer, which may be related to arsenic exposure [7]. Thus, a potential interaction effect between selenium and arsenic may have led to higher rates of T2D in the Se-supplemented group compared to the placebo effect [7]. However, this does not explain the differences between the observational and clinical trial results observed in this meta-analysis. Residual confounding is a potential explanation, as it is possible that there is a confounding variable that is associated with both the exposure (blood selenium levels) and the outcome (T2D) [31]. However, the work included in this systematic review generally controlled for potential confounders such as age, sex, body mass index, energy intake, and smoking status. Therefore, there would need to be another as-yet-unknown variable that influences both exposure and outcome. There could also be individual characteristics of participants, such as genotype, that influence any biological effects of selenium and require further study. 

Finally, the duration and timing of exposure to Se may be a key aspect of any effect on T2D. In the clinical trials, supplementation generally occurred for 3–5 years among older participants; observational studies may reflect longer-term, usual Se exposure through the diet. It is possible that lifelong consumption of Se is associated with risk for T2D while supplementation for a comparatively brief period is not. A primary challenge in examining nutrient relationships is determining whether the potential role is in the initiation, development, or exacerbation of disease or whether the variations in the observed nutrient levels are the consequence of disease sequelae. Additionally, nutrient effects may vary depending on the timing of inadequate or excessive nutrition. The SELECT and other selenium supplementation trials presumably examined the role of selenium as a strong antioxidant. However, as noted by Bellinger et al. [32], supplementation of selenium does not affect all selenoproteins equally and as previously discussed, choice within the supplemented form may also alter physiological uptake and use. In addition to its antioxidant properties, selenoproteins have a demonstrated effect in adipocyte differentiation [33]. This adipocyte differentiation effect may explain the increased T2D risk associated with dietary levels of selenium that presumably would have been similar during early formative years, whereas selenium supplementation during the later years of living may not play the same physiological role. 

The strengths of this work include its comprehensive approach to review and meta-analyses and the inclusion of the most recent data for Se and T2D. We carefully followed the PRISMA guidelines for data collection, including the registration of our study with the PROSPERO International prospective register of systematic reviews. In addition, we assessed study quality before incorporating data into the meta-analysis, and also contacted authors for any missing data. However, the limitations of this study must also be addressed. First, there was significant variability in the study populations and designs, including the exposure definition used in the studies we found. Measurement of selenium exposure varied among the observational studies comprising dietary intake or biochemical assessment including serum, plasma, and whole blood. These factors may have contributed to the substantial heterogeneity found among the observational studies. Further, pooled estimates were unadjusted; therefore, uncontrolled confounding may have biased the observational pooled estimate.

## 5. Conclusions

The effects of T2D may be briefly described as inadequate secretion and/or sensitivity to insulin. Selenoproteins are critical physiological antioxidants, able to exert insulin-like properties that in excess may impair insulin signaling [32]. Additionally, pancreatic beta cells express selenoproteins, providing biological plausibility that selenium holds a role in T2D. However, the findings from this meta-analysis indicate consistent moderate associations only between high levels of dietary or serum selenium and prevalent T2D and inconsistent results among studies aimed at assessing incident T2D. Our analysis demonstrates no consistent evidence that Se supplementation plays a role in T2D development among adults. 

## Figures and Tables

**Table 1:** The 15 manuscripts that met the *a priori* criteria for inclusion.

Author, Year	Study Population	Exposure Mean (SD) µg/L	Covariates	Key Findings RR (95% CI)
Li et al. 2017 [18]	Jiangsu Province; 122 newly diagnosed cases, 429 matched controls	Plasma Se 16.4 (11.9) *	Age, gender, BMI, family history, smoking and drinking status	OR = 6.14 (3.01–12.51)
Hansen et al. 2017 [19]	The HUNT Study; 128 cases, 755 controls	Whole blood Se 101.2 cases 101.4 controls #	Age, sex, BMI, WHR, education, income, smoking, family history of diabetes	OR = 0.93 (0.50–1.74) *p*-trend = 0.907
Zhang et al. 2017 [20]	REACTION study; 510 cases, 1327 controls	Whole blood Se 210 (50) cases 200 (50) controls	Age, gender, BMI, insulin, SBP, DBP	OR = 2.69 (1.31–3.49) *p*-trend < 0.001
Lu et al. 2016 [21]	Taipei medical center; 303 cases, 544 controls	Serum Se 88.2 (21.2)	Age, gender, current smoking, current drinking, physical activity, waist circumference, HOMA-IR	OR = 3.79 (2.17–6.32) *p*-trend < 0.001
Simic et al. 2017 [22]	The HUNT3 Survey; 267 self-reported cases, 609 frequency-matched controls	Whole blood Se 102.3 cases 102.3 controls	Age, sex, BMI, WHR, first-degree family history of diabetes, smoking habits, area, education, economic status	OR = 1.13 (0.65–1.96) *p*-trend = 0.530
Kohler et al. 2018 [23]	Cross-sectional Selenium Trial, *n* = 1714	Plasma Se 143.6 (28.9) T2D 138.7 (27.2) No T2D	Age, sex, BMI, race, ethnicity, smoking, education, and dietary intake of energy, protein, carbohydrate, total fat, and total fiber	OR = 1.77 (1.16–2.71) *p*-trend = 0.007
Galan-Chilet et al. 2017 [17]	Cross-sectional Hortega Study, *n* = 1452 Prospective cohort, *n* = 1234, 13.2 years follow-up	Plasma Se 84.2	Age, gender, education, urine cotinine, smoking status, alcohol intake	OR = 1.97 (1.14–3.41) *p*-trend = 0.03 HR=1.80 (0.98–3.31) *p*-trend = 0.15
Wei et al. 2015 [24]	Cross-sectional in China, *n* = 5423	FFQ 43.51	Age, sex, education, employment, BMI, activity level, WC, HTN, drinking, smoking condition, energy intake, fiber intake, and nutritional supplementation status	OR = 1.52 (1.01–2.28) *p*-trend = 0.03
Laclaustra et al. 2009 [13]	Cross-sectional NHANES 2003-2004, *n* = 917	Serum 137.1 (19.9)	Sex, age, race, education, BMI, smoking, cotinine, postmenopausal status, vitamin/mineral supplements	OR = 7.64 (3.34–17.46) *p*-trend = 0.002
Bleys et al. 2007 [14]	Cross-sectional NHANES III, *n* = 8876	Serum 126.5 (1.0) T2D 125.7 (21.0) No T2D	Age, sex, race/ethnicity, education, family income, postmenopausal status, cigarette smoking, serum cotinine, alcohol consumption, physical activity, BMI, C-reactive protein, hypercholesterolemia, serum triglycerides, HTN, GFR, vitamin/mineral supplementation; intake of beta-carotene, vitamin C, vitamin E; serum levels of albumin, alpha-carotene, beta-carotene, beta-cryptoxanthin, lutein/zeaxanthin, lycopene, uric acid, vitamin C, and vitamin E	OR = 1.57 (1.16–2.13) *p*-trend = 0.03
Gao et al. 2014 [15]	Uppsala Longitudinal Study of Adult Men (ULSAM), *n* = 936 men, 20 years follow-up	Baseline Serum 75.6 (14.3)	Age at baseline, BMI, cigarette smoking, leisure time physical activity, education	OR = 1.06 (0.83–1.38) *p*-trend = 0.497
Stranges et al. 2010 [16]	HORmones and Diet in the ETiology of Breast Cancer (ORDET) Study, *n* = 7182 women, 16 years follow-up	Baseline FFQ 55.7	Age, education, menopausal status, BMI, smoking, alcohol intake, energy intake, saturated/polyunsaturated fatty acid ratio, animal proteins, total carbohydrates, and weight change (follow-up exam–baseline)	OR = 2.39 (1.32–4.32) *p*-trend = 0.005
Thompson et al. 2016 [26]	Selenium and Celecoxib Trial, *n* = 1640 men, 35.6 and 35.5 months follow-up in the placebo and Se arms, respectively	200 µg/day selenized yeast	Random assignment to celecoxib, aspirin, and clinic	HR = 1.25 (0.74–2.11) *p*-interaction age = 0.02 HR = 2.21 (1.04–4.67) for age at randomization ≥63 years
Lippman et al. 2009 [25]	Selenium and Vitamin E Cancer Prevention Trial (SELECT), *n* = 17,448 men in Se and placebo arms only, median 5.45 years follow-up	200 µg/day *L*-selenomethionine	Randomized controlled trial	RR = 1.07 (0.94–1.22)
Stranges et al. 2007 [3]	Nutritional Prevention of Cancer (NPC) Trial, *n* = 1202 men and women, mean 7.7 years follow-up	200 µg/day selenized yeast	Age, sex, BMI, smoking status	IRR = 1.50 (0.98–2.30) HR = 1.55 (1.03–2.33)

Abbreviations: BMI, body mass index; CI, confidence interval; DBP, diastolic blood pressure; FFQ, food frequency questionnaire; GFR, glomerular filtration rate; HOMA-IR, homeostasis model assessment of insulin resistance; HR, hazard ratio; HTN, hypertension; HUNT, li; IRR, incidence rate ratio; NHANES, National Health and Nutrition Examination Survey; OR, odds ratio; REACTION, Risk Evaluation of cAncers in Chinese diabeTic Individuals: a lONgitudinal study; RR, relative risk; SBP, systolic blood pressure;Se, selenium; WC, waist circumference; WHR, waist-to-hip ratio. * Median (Quartile Range) # Median.
